# The Statistical Evaluation of Treatment and Outcomes in Head and Neck Squamous Cell Carcinoma Clinical Trials

**DOI:** 10.3389/fonc.2019.00634

**Published:** 2019-07-12

**Authors:** Catherine Fortpied, Marie Vinches

**Affiliations:** EORTC Headquarters, Brussels, Belgium

**Keywords:** statistical design, statistical analysis, clinical trial, head and neck cancer, treatment, comorbidities, patient population, endpoint

## Abstract

The purpose of this article is two-fold: to help statisticians confronted with the design, implementation and analysis of clinical trials and new to the field of head and neck cancer; but also to sensitize research physicians with the role, the tasks and the challenges faced by the medical statisticians. These two purposes altogether will hopefully encourage and enable fluid communication between the research physician and the medical statistician and the understanding of each other's field and concerns. In particular, the methodological challenges resulting from the heterogeneity of the head and neck cancer, the complexity of the treatments and the associated comorbidities are presented with examples borrowed from medical literature and from the practical experience of the authors in this field.

## Introduction

Medical research and conduct of clinical trials is inconceivable today without statistical expertise. This is officially acknowledged in Europe since 1990 when the Committee for Proprietary Medicinal Products (CPMP) (1) adopted a Note for Guidance covering the subject of Good Clinical Practice (GCP). This note stated that “access to biostatistical expertise is necessary before and throughout the entire procedure, commencing with designing of the protocol and ending with completion of the final report.”

The purpose of this paper is two-fold: to help statisticians confronted with the design, implementation and analysis of clinical trials and new to the field of head and neck cancer; but also to sensitize research physicians with the role, the tasks and the challenges faced by the medical statisticians. These two purposes altogether will hopefully encourage and enable fluid communication between the research physician and the medical statistician and the understanding of each other's field and concerns.

According to the title of the paper, the first section explains what are the requirements to conduct a rigorous statistical analysis of a clinical trial: in particular the predefined analysis plan, the data, and the software. Then moving to the field of head and neck cancer, we will explain how the heterogeneity of the disease, the multimodality nature of treatments and the associated comorbidities influence the methods used to design and analyze clinical trials in this field. Key methodological and statistical concepts are explained and illustrated with examples borrowed from medical literature and from the practical experience of the authors.

## What Makes a Statistical Analysis a Good Statistical Analysis?

### Pre-defined Analysis Plan

Different statistical approaches can lead to different numerical results and hence influence the interpretation of the trial. The statistician will need to choose among the different possible statistical methods. The choice will depend on the nature of the data (e.g., continuous, binary, categorical, or time-to-event), the underlying assumptions about the statistical distribution (e.g., non-parametric, semi-parametric, or fully parametric), and the amount of data (e.g., asymptotic methods for large samples or exact methods for small samples). A demonstration of this, although based on observational data outside the medical field, is provided in the confronting article by Silberzahn et al. (2), which reports how different analytical methods can lead to different results. Lack of prespecification may affect the trial's validity by allowing the researchers to consciously or unconsciously select the analysis approach that provides the most favorable results. It is therefore important that these decisions are prespecified before seeing the trial data. This is why, and as stated in International Conference on Harmonization (ICH) E9 guideline (3), the study protocol must include the main features of the data analysis: definition of the analysis populations, timing of interim and final analyses, precise definition of the study endpoints, methods used for estimation, confidence intervals, and hypothesis testing; adjustment of significance and confidence levels; subgroup analyses. In addition, with the pre-specification of the analyses and more specifically of the hypotheses tests, the extent of multiplicity is clearly stated (multiple endpoints, multiple comparisons of treatments, repeated evaluations over time, interim analyses) and measures to control the risk of overall Type I error, i.e., of false positive findings, can be taken. Unplanned analyses are sometimes conducted. For example, when new questions based on the observed data emerge or when heterogeneity of the treatment effect across subgroups of patients needs to be assessed. When reporting and publishing the results of a clinical study, as stated in Consolidated Standards of Reporting Trials (CONSORT) guidelines (4), results from these *post-hoc* analyses have to be clearly distinguished from the results of the preplanned analyses. As the former cannot lead to firm conclusions, they are solely considered as exploratory and hypothesis generating. To ensure the completeness and the appropriateness of the written statistical analysis plan, a second statistician should ideally validate it.

### The Clinical Data

Accuracy, consistency, completeness and reliability of the clinical data is obviously critical for the analysis and interpretation of the study. Data management processing involves several steps and usually a high number of actors. The design of the case report forms and the clinical database are developed by the central data manager. The reporting of each patient data is done by the investigators from source documents. The interactions between the local investigators and the central data manager allows the verification and correction of the data and the traceability of the data flow. Principles established in GCP (5) and the sponsor's standard operating procedures should constitute a safeguard against poor data management.

### Computer Software Validity

Based on the statistical analysis plan, the statistician will process the data statistically. He/she will produce a descriptive analysis in the form of tables and graphical displays and an inferential analysis consisting of estimated effect sizes, their precision (such as 95% confidence interval) and significance (*p*-values). The credibility of the numerical results of the analysis depends on the quality and validity of the softwares used, either externally or internally written (3). A validated software and programming language, such as used in the Statistical Analysis System (SAS^®^, Cary NC, USA) should be used to produce the statistical analysis outputs. The study statistician should develop the statistical analysis programs specific to his/her study using built-in SAS procedures and in-house programmed SAS macros that automate repetitive data processes. To ensure the correctness of the results, a second, independent statistician should validate the analysis program, by independent programming, at least for the analysis of the primary and key secondary endpoints of the study.

### From the Statistical Analysis to the Publication

The role of statistics is to translate information into knowledge, which is, according to the renowned statistician Stephen Senn, the challenge that faces statisticians (6). When browsing the Royal Statistical Society website (7), one can read that the medical statistician will see his/her work “influence clinical practice, help guide public health education and policies, or add to current knowledge, sometimes leading to further research studies.” It is not enough to produce statistical outputs, statistical judgment must also be exercised for the interpretation and presentation of the results. The statistician will make sure that the conclusions are presented or disclosed in a manner that fairly reflects the evidence supported by the results. In this regard, we would like to caution about the use or overuse or even misuse of *p*-values: not only because the concept of the *p*-value is often misunderstood but also because it is not a substitute for medical judgement. *P*-values should not be provided alone but should be accompanied with effect sizes and their precision in order to be able to assess the clinical relevance of the results (8, 9).

All research results should be published, irrespective of the findings (both positive and negative, statistically significant, or not). As stated by Tam et al. (10), “Non publication of clinical trials breaks an implicit contract with trial participants, institutional review boards, and study sponsors and society in general.” As a measure to prevent publication bias, the International Committee of Medical Journal Editors (ICJME) (11) recommends editorial decisions not to be driven by the clinical trial results but by the originality, the quality and the contribution to scientific knowledge. Furthermore, regulatory bodies, Food and Drug Administration (FDA) and European Medicines Agency (EMA), have initiated moves toward greater transparency by requesting that the aggregated results of (drug) clinical trials are disclosed in public domain to US ClinicalTrials.gov (since 2007 and then extended to non-licensed products in 2017) (12) and/or to EMA European Clinical Trials Database—EudraCT (since 2014) (13). Keeping abreast of statistical methodology.

Again quoting the Royal Statistical Society website (7), the medical statistician is also “part of an academic group which develops statistical methodology to be applied to medical research.” Up to the nineties, the methodology for the design and statistical analysis of clinical trials was well-established and statisticians could do their job with a limited number of tools. Since then, parallel developments, such as increased computer power, advances in biology science leading to the emergence of a new class of treatment called “targeted,” brought new opportunities for clinical development and new methodological challenges (14). Some of these methodological challenges are discussed below in the context of head and neck cancer clinical studies.

### The Medical Statistician: From the Start, in Close Interaction With the Research Physician, the Data Manager and the Clinical Operations Team

A statistical analysis can only be translated into knowledge if the study has been adequately designed to answer the key research questions of the study. The medical statistician develops an appropriate design that will ensure the trial to provide the answer to its objectives within the limits of existing statistical methodology, starting from the rationale, the objectives and the clinical background defined by the research physician. The designs consists of the statistical and methodological setup of the trial, including elements such as randomization, stratification, planning of the statistical testing of primary and secondary endpoints, adjustment of those comparisons for covariates, sample size calculation taking into account type I and type II errors. It is at this point that the statistician and the research physician need to interact closely. Discussions should identify the practical constraints of the study, particularly in terms of potential accrual and overall study duration. With these elements in hand, the statistician will propose a statistical design for the study. Several options are typically discussed before a final design is agreed upon.

In addition, as the statistician is typically concerned by bias and precision, he/she is not only involved in the pure statistical aspects of the study. But he/she will also participate in planning operational aspects that may potentially induce a bias or undesired variability affecting the interpretability of the study results. As such, the statistician carefully reviews the procedures planned for the study such as selection, diagnosis and staging of patients; treatment administration; follow-up assessments; data processing (3). He also pays attention to the potential aspects of the protocol where adherence is more difficult to achieve in order to minimize the incidence of violations of the entry criteria, non-compliance, withdrawals, losses to follow-up, missing data and other deviations from the protocol. Deviations may affect the subsequent analyses and ultimately the interpretation and conclusions of the study.

The medical statistician is thus involved from the start up to the end in a study life cycle. Table 1 provides a synthetic list of his/her responsibilities.

**Table 1:** Responsibilities of the trial statistician in a study life cycle.

**Study life cycle**	**Responsibilities of the trial statistician**
Study design	Contributes to the definition of the study objectives and to the selection of the primary and secondary endpoints Proposes one or several statistical designs until final selection Computes the sample size and the study duration For randomized studies, specifies the method and parameters of the randomization procedure Defines accurately the trial endpoints and the methods of assessment Designs the early stopping rules and interim monitoring plan Writes the statistical analysis plan
During the study conduct	Monitors the assumptions underlying the statistical design Interacts with the Independent Data Monitoring Committee, when pre-specified in the study protocol or in case of unanticipated issues Contributes to the amendment of the study protocol in case of major changes to the statistical considerations of the trial
At each interim, final or long-term data analysis	Assesses the quality of the clinical database for the intended analysis, in terms of completeness and consistency Develops the analysis programs to produce the required tables, graphical displays and inferential analyses Writes the statistical analysis report Contributes to the presentation and publication of the study results Submits the final study results to EMA European Clinical Trials Database (EudraCT) and to US ClinicalTrials.gov public websites

## The Specificities of Head and Neck Cancer and Statistical Considerations

### Heterogeneity of the Disease

Head and neck cancers are a group of diseases characterized by phenotypic, etiological, biological and clinical heterogeneity. Squamous cell carcinoma is the predominant histology type (15). The complexity of the upper respiratory and gastro-intestinal apparatus creates a number of anatomical subdomains that are apprehended together. Still the prognosis is specific to each localization, correlated to a distinct TNM classification (16). Historically, the most common risk factors are tobacco and alcohol consumption, responsible for up to 75% of head and neck squamous cell carcinoma (HNSCC) (17). The etiologic association of human papillomavirus (HPV) with a distinct subset of HNSCC that occur mostly in oropharynx is increasing, affecting non-smokers in developed countries. HPV positive oropharyngeal tumors have better survival, particularly for locoregionally advanced disease (18). The comprehensive genomic analysis performed by The Cancer Genome Atlas (19) revealed the genomic heterogeneity of this disease with clear differences between HPV negative and positive tumors. This, in the era of personalized medicine, could lead to different treatments depending on the plausible therapeutic targets.

#### Selection of Patient Population

A clinical research question is intrinsically defined in terms of a specific population. Eligibility criteria, assumptions about the prognosis of patients and the magnitude of the treatment effect in the intended population, stratification of patients within the clinical trial are all fundamental questions when planning a clinical trial (20). These decisive elements of the design can be obtained from expert opinion and a careful review of the medical literature.

Past and currently ongoing clinical studies define their target population based on the anatomical location of the disease, the classical TNM classification and more recently on the distinction between HPV positive and negative nature of the disease. In addition, a search of medical literature reveals an abundance of articles reporting the assessment of prognostic factors in head and neck cancer and the classification of patients according to different risk levels of progression, recurrence, or death. These analyses are useful to circumscribe the population of interest for our research question and when searching Medline for head and neck risk classification (see Supplementary Material for the exact Search query used), one retrieves 404 papers published in the past 10 years, 59 of them in 2018. But are all these analyses conducted adequately from a methodological point of view? Are the conclusions useful from a clinical point of view (21)? How can we separate the wheat from the chaff? The statistician will do his/her best to review these articles with a critical eye to evaluate the methodology used, and to assess how applicable and generalizable these results are. More specifically the statistician scrutinizes multiple aspects of the work, including but not limited to: characteristics of patients included in the modeling; selection and definition of the outcome of interest (locoregional failure, risk of distant metastasis, or survival); treatment received by the patients included in the analysis; set of candidate prognostic factors; data analysis method (statistical model such as Cox model or machine learning tools such as neural networks or random survival forests); model performance measures; internal and external validation procedures (22).

An example of the complexity to define the targeted patient population can be illustrated by the European Organization for Research and Treatment of Cancer (EORTC) trial 22931 (23) and the Radiation Therapy Oncology Group (RTOG) trial 9501 (24). Both trials evaluated post-operative chemoradiation vs. radiation alone in patients at high-risk of recurrence after surgery. The definition of high-risk and therefore the inclusion criteria, although sharing some common criteria, differed between the two studies. While the eligibility criteria common to both trials were the presence of extracapsular extension and/or microscopic-sized tumor involvement of the surgical margins of resection, some differed. The EORTC study included in its selection of risk factors stage III/IV disease, the presence of enlarged lymph node(s) at level IV or V in patients with oral cavity or oropharynx carcinomas, pathological demonstration of vascular embolisms, and/or perineural disease. The RTOG study included in its selection of risk factors the presence of tumor in two or more lymph nodes, as was suggested by the analysis of the RTOG database. In 2004, the publication of the two studies established, with level I evidence, that concurrent chemoradiation was more efficacious than radiation alone as adjuvant postoperative treatment, in terms of local-regional control and disease-free survival. Because of the difference in the definition of “high risk” features between the two trials, additional analyses were conducted to identify precisely which patients were more suitable for such intense treatment (25). The findings suggested that microscopically involved resection margins and extracapsular spread of tumor from neck nodes were significant prognostic factors for poor outcome. Despite the limitations inherent to a retrospective subgroup analyses, their results are now the basis for the selection of patients in clinical trials in the postoperative setting (e.g., EORTC study 1735: NCT03673735).

Another example of clinical trials based on a risk classification is given by an ongoing Canadian Cancer Trials Group study (NCT03410615), testing the effect of immunotherapy in intermediate-risk, HPV-positive, locoregionally advanced, oropharyngeal squamous cell cancer. Here the definition of intermediate-risk is based on data showing that HPV-related oropharyngeal cancer patients with limited neck disease (N0-N1) have a favorable prognosis, even without chemotherapy (26, 27).

#### Favorable Prognosis and Non-inferiority Designs

The majority of studies test novel treatments or combination of treatments in order to improve disease outcome and survival in patients with unfavorable prognosis, with intermediate or high risk of progression, recurrence or death. Some trials are also designed and conducted in patients with a favorable prognosis, in order to assess whether treatments reduce acute and late toxicity while preserving a similar disease outcome and survival.

Since the identification of HPV positive patients as a separate disease entity with a more favorable prognosis, a number of studies have been developed to de-intensify treatment in these patients. This is the case of RTOG 1016 study (NCT01302834) (28) and De-Escalate study (NCT01874171) (29). Both studies attempt to replace cisplatin by the epidermal growth factor receptor (EGFR) inhibitor cetuximab in patients with HPV positive oropharyngeal cancer. The objective of these studies is to maintain a similar patient survival while reducing toxicity, and as such they require a different type of design. RTOG 1016 was designed as a classical non-inferiority trial with overall survival as primary endpoint. One point of consideration for non-inferiority studies is the value of the non-inferiority margin that is considered as an acceptable loss, in disease outcome or survival in view of the gain in toxicity. It has to be put in perspective with the prognosis of patients since a loss of 10% does not mean the same when survival rate with the standard of care is at the level of 70 or 90%. This non-inferiority margin needs to be small enough to be considered as non-clinically relevant and certainly substantially lower than differences targeted in superiority trials (30). Defining the non-inferiority margin is an essential part of a non-inferiority design just as the difference is essential to the design of a superiority or difference trial. Both have to be pre-defined based on clinical and statistical considerations and both have a strong impact on the sample size, the trial duration and its cost. The primary endpoint of RTOG 1016 was overall survival and the study was designed to reject the null hypothesis of inferiority, with a non-inferiority margin of 9%, meaning that a decrease of 9% in 5 years overall survival was considered, by the RTOG 1016 team, acceptable. By contrast, the primary endpoint of De-Escalate study was overall severe (grade 3–5) toxicity events at 24 months from the end of treatment and the study was powered to detect a reduction in the rate of severe toxicities in cetuximab arm compared to cisplatin arm. Equivalent disease control and survival between treatment arms were hypothesized but the study was not formally powered to show non-inferiority. Interestingly, overall survival and time to recurrence were planned to be compared between the two arms using the log-rank test, which is a test aiming at detecting differences, not aiming at showing non-inferiority or equivalence; now, failing to detect a difference does not mean there is no difference as “absence of evidence is not evidence of absence.”

#### Designs for Studies in Rare Cancers and Accrual Issues in Randomized Trials

With a heterogeneous disease, distinct subtypes, in terms of tumor localization and biological characteristics, need to be investigated separately in small groups of patients. In case of a rare population, a classical design may just not be feasible and we need to reflect on the level of evidence we still wish to reach (31). One possible solution is to allow more uncertainty, that is to allow a Type I error higher than the traditional 5% two-sided that is required to reach scientific evidence for a superiority trial and/or to allow a Type II higher than the classical 10 or 20% which is equivalent to say that the study is only powered to detect large differences. In these cases, we need to be careful with the consequences of relaxing the errors/power, given that it is unlikely that another trial will be conducted to confirm the results. EORTC study 1206 (NCT01969578) aims to assess the superiority of androgen deprivation therapy (ADT) over standard chemotherapy (CT) in patients with recurrent/metastatic salivary gland cancer. It has been designed with a one-sided Type I error of 10% and with a power of 80% to detect an ambitious difference between ADT and CT in progression-free survival with a hazard ratio HR = 0.56. Because our predictions indicate that the study will likely fail to recruit the total planned number of patients in a reasonable timeframe, the design has been revised recently. An analysis of the primary endpoint from a non-inferiority perspective has been added, by pre-specifying a non-inferiority margin, in case the study fails to meet the criteria of superiority. This non-inferiority test has adequate statistical power under the hypothesis of the superiority of ADT over CT. If the objective of non-inferiority is met, this is considered valuable from a clinical point of view given the favorable safety profile of ADT compared to CT, except for sexual dysfunction, and the dismal prognosis of these patients.

EORTC study 1206 is one of the studies developed within the International Rare Cancers Initiative (IRCI) (32), a strategic collaboration between several academic organizations, including EORTC. IRCI's aim is to stimulate and facilitate the development of international clinical trials for patients with rare cancers. Some of the studies from the IRCI portfolio, focusing on rare cancers, are designed using Bayesian methodology. With this methodology, the conclusion of the study is based on the combination of the study data itself together with prior knowledge based on literature review, previous studies, meta-analyses or the elicitation of expert's opinion. Contrary to the classical (frequentist) approach, the focus of the Bayesian approach is on estimation rather than testing hypotheses, with data being used to reduce uncertainty about the size of the treatment effect. However, we remain unsure regarding the advantages of this methodology as in a small trial the choice of the prior may carry heavy weight thus influencing the final results. Moreover, in rare cancers only weak prior evidence might be available. In addition, in absence of prior information, Bayesian designs do not immediately add value over equivalent classical (frequentist) designs in terms of the statistical properties (type I error rate, type II error rate, power, sample size). However, this does not prevent that a trial designed in a frequentist setting is analyzed using Bayesian methods, and the results interpreted using the posterior distribution of the treatment effect which is obtained from the combination of prior knowledge with currently observed trial data. To our knowledge, only one prospective clinical trial in head and neck cancer is designed using a Bayesian methodology: this is two-stage phase II design of Magnetic Resonance-guided radiotherapy dose adaptation in patients with HPV positive oropharyngeal cancer. This study uses Bayesian decision rules applied to loco regional control and toxicity to make the go/no-go decision for each stage (33).

Beyond the specific case of a rare disease, accrual of patients in the clinical trial may be slower than initially expected as a result of strict eligibility criteria, over-optimism of participating institutions at the start of the study; lengthy approval of the study by competent authorities and ethic committees; patients reluctant to enter the study or to be assigned a treatment at random. Slow accrual leads to longer study duration and therefore delayed availability of the study results, possibly when the main research question posed by the study is no longer relevant given the evolution of clinical research in the field. In order to speed up accrual, some actions are envisaged such as broadening the eligibility criteria of the study; opening the study to additional treating institutions, countries, or other research organizations. In some other cases, the ultimate decision is to close the study definitely before having reached the targeted sample size. It is then necessary to evaluate to which extent the available data can be used to assess the objectives of the trial. Sometimes the available study data allows to conduct the initially planned analyses with a decreased but still acceptable statistical power, say 70% instead of the initially stated 80%. When data are scarcer, only a mere descriptive analysis of the study data is feasible. In other cases, it is possible to rescue the study through a substantial revision of the statistical analysis plan. To ensure the validity of such a revision, it should be done before any of the data is revealed.

#### Treatment Allocation

These considerations about the heterogeneity of the disease are at the basis of the selection of the study population but also of the stratification of patients within the randomized clinical trial. This stratification is to be taken into account not only in the process of randomization, in order to produce comparable treatment arms in terms of factors that affect the course of the disease, but also at the data analysis levels. Due to the association between these factors and the outcome variable, adjustment for such factors generally improves the efficiency of the analysis.

Randomization tends to produce treatment arms in which the distributions of prognostic factors, known and unknown, are similar (3). Achieving a balanced allocation overall and for important prognostic factors allows to attribute differences in outcomes to differences in efficacy of the treatments under study; this is the concept of causality. In randomized studies, the most relevant factors for stratification need to be identified, bearing in mind that too many stratification factors are detrimental to a balanced allocation. The number of stratification factors and which ones to select is discussed between the research physician and the medical statistician until a compromise is found. It is particularly important to consider institution as a stratification factor in order to account for the differences across treating sites in terms of patient selection, treatment and care, assessments, and data reporting. In EORTC study 1420 “Best-of” (NCT02984410), a randomized phase III study the main objective is to assess the patient-reported swallowing function over the first year after treatment start with either Intensity-Modulated Radiation Therapy (IMRT) or Trans Oral Surgery (TOS) among patients with early stage oropharyngeal, supraglottic, or hypopharyngeal carcinoma. In this study, the eight disease localizations were classified into two strata, lateral vs. central lesions, thought to be an appropriate classification taking into account that the primary endpoint was the swallowing function. Two other clinical stratification factors were considered, N stage and the swallowing function score at baseline. Because of the relative small size of the study, 170 patients potentially accrued in more than 30 treating institutions from 8 countries, stratifying by treating institution would have resulted in too many small strata and in this study the decision was made to group them by country.

For each study, the statistician evaluates which treatment allocation method is most appropriate. The two most common methods are the static permuted blocks method and the dynamic minimization algorithm (34). While the minimization method is often discouraged by Regulatory Authorities due to theoretical concerns, a cancer-specific review published in 2010 (35), indicated that it becomes more common over time and is used more frequently when an academic cooperative group is involved. For both methods, it is recommended to perform computer simulations to assess the performance of the chosen method and the stratification design, in terms of the balance of the stratification factors over the treatment arms.

#### Subgroup Analyses

It is tempting to conduct multiple subgroup analyses in large studies of patients with heterogeneous characteristics. However, as indicated in ICH E9 and often reiterated in medical and statistical literature, such analyses carry the risks of generating false positive findings due to statistical testing in multiple subgroups. It also runs the risks of false negative findings due to the small size of the subgroups. The appropriateness of the use and interpretation of subgroup analyses on the basis of the CONSORT statement requirements (4) was investigated in 188 phase III randomized controlled trials in solid tumors, published between 2011 and 2013 (36). When focusing on the 102 articles claiming a subgroup difference, for 24% of them it was unclear whether the subgroup analyses were prespecified or *post-hoc*, and subgroup analyses of 36% of these trials were *post-hoc* only. Eighty-four percentage of these trials reported more than five subgroup analyses but only 6% cautioned about multiplicity. This review shows that despite recommendations from the CONSORT statement published more than a decade ago, the reporting of subgroup analyses is generally not adequate to provide valuable information in guiding clinical decisions.

It is worth emphasizing that comparing outcomes in patients subgroups defined by some other outcomes or variables measured after treatment start, such as dose intensity, compliance to treatment or adverse events require non-standard analysis methods, as these variables are themselves affected by treatment (37, 38). In particular, standard analysis methods of comparing survival between responders with non-responders are wrong and lead to biased estimates and misleading conclusions. This bias results from the fact that responders must live long enough for a response to be observed and that patients who die early without observing a response are automatically classified as non-responders. A better approach, proposed by Anderson et al. (37), is the landmark method, where each patient's response is determined at some fixed time point after treatment start and the survival estimates are calculated from that time point. This method has for example been applied in the analysis of a study of induction treatment followed by chemoradiation in advanced stage in head and neck cancer. An 8 weeks landmark analysis was carried out to compare survival between patients with positive vs. negative biopsy of the primary site done after induction. A 4 months landmark analysis was also performed to evaluate the effect of maintenance therapy on survival. Survival was computed from the landmark (39). Similarly an analysis of the predictive value of cetuximab-induced skin toxicity in recurrent or metastatic head and neck cancer was conducted using the landmark method applied to PFS and OS counted from 90 days after the start of therapy (40).

When dealing with subgroup of patients, and especially in the era of personalized medicine, the question whether some patient characteristics or some biomarkers are predictive of treatment benefit is of interest. To determine whether a biomarker is potentially predictive, a formal and adequately powered statistical test of the treatment-by-biomarker interaction needs to be performed (41). For more detailed considerations on the statistical methodology required to establish predictive biomarkers, readers are referred elsewhere (42). To date, in the field of head and neck cancer, no biological marker has been proven to be predictive (43).

### Complexity of the Treatments—Multimodality

Treatment for head and neck cancer is complex and is based on different levels of evidence as stated in the National Comprehensive Cancer Network guidelines (44). Treatment options depend on the stage of the disease: early, locally advanced or recurrent/metastatic. Surgery, radiotherapy, chemotherapy and targeted therapy are all front line options, alone or in combination, depending on the tumor characteristics and stage of the disease. New categories of treatment have been evaluated in head and neck cancer, check point inhibitors have been approved in the metastatic settings with improved survival in first [pembrolizumab (45)] and second line [nivolumab (46)]. With new treatments available, new combinations are being tested. Still multimodality remains key.

#### Selection of Activity/Efficacy Endpoints of Interest

The therapeutic effect of a new treatment or combination of treatments is assessed by means of endpoints selected according to the study objectives.

In early phase studies, the main endpoint is usually selected to capture the effect of the treatment on the tumor, that is whether the treatment is expected to induce a complete disappearance of the tumor, shrinkage of the tumor or a stabilization of the disease. Complete response (CR) or response (complete response CR or partial response PR), has long been selected as primary endpoint of early phase studies. However, other endpoints may be preferred, such as disease control (complete response CR or partial response PR or stable disease SD) to evaluate treatments with a mechanism of action different from chemotherapy, such as targeted or immunotherapy, or where the response to treatment is difficult to assess. Progression free survival (PFS) rate or another time to event endpoint (TTE) evaluated at a fixed point in time after randomization or start of treatment may also be used so the timing of the final analysis is fixed and not dependent on a pre-specified number of events to be observed. When designing a study to evaluate treatments that induce disease stabilization rather than disease reduction, it is recommended that the study includes an internal control arm to make sure the effect of treatment is not confounded with the natural course of the disease. This is especially important if historical information on the control treatment is lacking or limited due to differences in patient population (e.g., biomarker selected population), in staging system, in imaging / diagnostic tools for assessing outcome, etc. The EORTC study 1559 [NCT03088059 (47)] is an umbrella trial (48) with a platform for enrollment, screening and central profiling of patients who are subsequently allocated to one of the molecularly defined sub studies and treated with a matched experimental treatment. Different designs are used across the study, reflecting differences in study objectives and in the mechanism of action of the investigated treatment among the sub studies: in particular, a single arm design with response as primary endpoint is chosen for some sub studies, while others are designed as a randomized two-arm trial, with physician choice as control treatment and with progression-free survival assessed 4 months after randomization as primary endpoint.

Some early phase studies are designed with the objective to assess the feasibility of the treatment, in which case the main endpoints may be defined as the proportion of patients completing therapy, the rate of patients without severe toxicity, the rate of patients compliant with protocol treatment or similar endpoints. This is the case of the EORTC study 24061 (49), a randomized phase II feasibility study of cetuximab combined with 4 cycles of Docetaxel, cisplatin, and fluorouracil (TPF)followed by chemoradiation with platinum. The main objective of this study was to select the platinum compound, cisplatin or carboplatin, for the chemoradiation regimen to be evaluated in a future Phase III study. Unfortunately, the study was closed prematurely for safety reasons.

In phase III studies, overall survival (OS) remains the gold standard for the demonstration of clinical benefit, as it is an objective and accurate measure, its importance is unquestioned and it addresses both safety and efficacy. Because overall survival analysis requires a large sample size and may require long follow-up, the investigators may power the study for an alternative endpoint. Doing so, it reduces study timeframe, and improves study feasibility while still capturing a clinical benefit relevant for the patient. Alternative endpoint may be time to local or loco-regional recurrence/progression for early stage disease or to evaluate a local therapy (e.g., EORTC study 1219, NCT01880359); disease free survival in the adjuvant setting [e.g., EORTC study 1735, NCT03673735 or LUX-Head & Neck 2 (50)]; or progression-free survival in the advanced setting [e.g., LUX-Head & Neck 1 (51)]. In 2009, Michiels et al. (52) showed that progression-free survival, defined as the time from randomization to locoregional relapse, distant recurrence, or death whichever comes first, can be used as a surrogate endpoint for overall survival to assess the treatment effect of radiotherapy and chemotherapy in randomized trials of locally advanced HNSCC. The surrogacy has been established based on [1] the individual-level correlation between Event Free Survival (EFS) and OS, and [2] the correlation between treatment effects on EFS and OS following the methodology developed by Buyse et al. (53). However, we need to remember that, as pointed out by Michiels et al., EFS is a surrogate endpoint for OS only for chemotherapy or radiotherapy, but cannot be assumed for immunotherapy and for targeted agents, which have a different mode of action. We will come back later on this matter.

#### Definition of Endpoints

It is not enough to select the endpoint of interest but we also need to state how exactly it is defined and how it is assessed.

International standards are available for measuring response in clinical trials, the most common being the Response Evaluation Criteria in Solid Tumors (RECIST) (http://www.eortc.org/investigators-area/recist). Because of the loss of information inherent to categorizing a continuous measure of tumor shrinkage into categories (progression, stable, response), more and more often waterfall plots are used to display graphically the individual numerical change in tumor size for all patients.

Time to event endpoints need to be defined very clearly and it is very useful that their exact definition is accurately provided in the scientific publications, as there is considerable heterogeneity in the literature regarding these definitions (54). The methodology section should describe which events are of interest for the selected endpoint, which events constitute competing risks, which events are censored and which events are ignored. In a complex disease such as head and neck cancer with multimodal treatments, for each time-to-event endpoint other than overall survival, and depending on the setting, the following events need to be considered: residual disease after curative treatment, local, regional or distant progression, second primary cancer, death due reasons other than progression. To date there is no consensus on how these and other events such as elective neck dissection and salvage surgery (with residual disease detected or not, depending on the timing of these procedures) are taken into account in the definition of endpoints. It is the purpose of the Definition for the Assessment of Time-to-event Endpoints in CANcer trials (DATECAN) project (55) to reach, by consensus among experts, a standardization of the definitions of commonly used time-to-event endpoints in cancer clinical trials. In addition, events such as treatment stop or switch before the event of interest being reported are not handled the same way by all methodologists, as some recommend ignoring the treatment switch while others recommend censoring these cases (56). The latter approach is highly problematic since it ignores the issue of informative censoring and is not recommended by the EMA (57) while it is proposed in FDA guidelines (58, 59).

In EORTC study 1219 (NCT01880359), a blind randomized multicenter study of accelerated fractionated chemo-radiotherapy with or without the hypoxic cell radiosensitizer nimorazole, the primary efficacy endpoint is time to locoregional recurrence. This is counted from the day of randomization to the day of first record of appearance of local or regional progression, assessed via clinical, imaging or pathological exam. Distant recurrence/progression and second cancers diagnosed before locoregional recurrence and death in absence of locoregional recurrence are not considered events of interest. But these events are considered as competing risk events in the analysis of the primary endpoint, because they may alter or even preclude the onset of locoregional progression. Therefore, during the design phase, the statistician together with the research physician needs to make assumptions, not only regarding the risk of locoregional progressions but also the risk of distant recurrence/progression, second cancers and death in absence of locoregional recurrence. It is also recommended to monitor these assumptions regularly as they have the potential to directly impact the sample size of the trial, the timelines for the analyses and possibly the statistical power. When a marked departure from the original statistical design assumptions, such as the ones described above, is observed, the consequences need to be evaluated as well as the need for a modification of the study design. In order to maintain trial integrity, an Independent Data Monitoring Committee (IDMC) is consulted and the study design is revised, based on the IDMC recommendations, by a statistician not directly involved in the conduct of the study.

Schedule of disease assessments plays a critical role in the evaluation of endpoints. The assessments should ideally match standard practice but for the purpose of the clinical trial they should be planned adequately to capture the effect of treatment. In a multi-arm study, there should also be symmetry between treatment arms in order not to introduce a bias in the comparison of the treatments. With time-to-event endpoint other than overall survival, such as progression-free survival, the exact time of progression is unknown and progressions that occur in between visits are commonly assigned to the visit at which progression was detected. This leads to an over-estimation of the time to progression and a loss of statistical power (56). The analysis may become problematic and biases may arise when clinic visits are missed or delayed. In some cases, it is a challenge to reach a common schedule of assessment across arms because of the intrinsic difference between treatments: surgery vs. radiotherapy as in EORTC study 1420 “Best-of” (NCT02984410), chemotherapy vs. targeted agent as in EORTC study 1206, induction chemotherapy vs. no induction. When the schedules cannot be made symmetrical across arms, the time assessment biases inherent to the trial may be taken into account by the statistical analysis, by assigning the progressions or recurrences to a specific point in time (e.g., the next planned visit). This technique was used in a study comparing three nonsurgical treatment strategies to preserve the larynx in patients with locally advanced larynx cancer (60). Patients were randomized between induction cisplatin/fluorouracil followed by radiotherapy, concomitant cisplatin and radiotherapy, or radiotherapy alone. The primary endpoint was a composite endpoint of laryngectomy-free survival. In this study, to account for differences between treatment arms in the timing of protocol-specified disease assessments, patients with recurrence or censored before 6 months after random assignment were counted as having treatment failure or censored at 6 months, for efficacy endpoints other than overall survival.

#### Definition of Analysis Populations

If all subjects enrolled into a clinical trial satisfied all entry criteria, completed treatment, followed all trial procedures perfectly with no losses to follow-up, and provided complete data records, then the set of subjects to be included in the analysis would be self-evident. But, in practice, it is doubtful if it can ever be fully achieved specially in the setting of a life-threatening disease, when dealing with complex treatments, administered concomitantly or sequentially.

The intention-to-treat principle requires that the primary analysis should include all enrolled subjects. In many clinical trials, this principle provides a conservative strategy and estimates of treatment effects that are more likely to mirror those observed in subsequent practice (3). However, in specific cases, such as early phase trials, the primary analysis is conducted in the per-protocol population, that is, in the subset of patients who are more compliant with the protocol in order to maximize the opportunity for a new treatment to show activity. For trials with a non-inferiority objective, it is recommended to conduct the main analysis on the per-protocol population in addition to the intention-to-treat population as the latter one may be biased toward demonstrating non-inferiority. It is to be noted that the per protocol analysis may lead to biased results when adherence to the study protocol is related to treatment and outcome.

A textbook example of such bias is given by study TTCC 2002 (grupo español de Tratamiento de Tumores de Cabeza y Cuello), a randomized phase III trial comparing induction chemotherapy followed by chemoradiotherapy vs. chemoradiotherapy alone as treatment of unresectable head and neck cancer (61). The intention-to-treat analysis including all randomized patients showed no advantage of induction chemotherapy followed by chemoradiotherapy over chemoradiotherapy alone; while the analysis excluding patients from the induction arm who did not reach the chemoradiotherapy part of the study resulted in a benefit in favor of the induction arm. The latter analysis, which was the one first published (62), was obviously biased because of the selection of the “best” patients from the induction arm.

#### Immunotherapy: Impact on Trial Endpoints

With the advent of immunotherapies, because of the different mechanism of action, how efficacy/activity endpoints are defined and evaluated poses a number of new methodological challenges (63).

Novel criteria for the evaluation of antitumor responses with immunotherapeutic agents were first developed and published in 2009 by Wolchok et al (immune-related response criteria:irRC) (64), as an attempt to capture new response patterns observed with immune therapy in advanced melanoma beyond those described by RECIST. These criteria, based on bidimensional measurements, were adapted in 2013 by Nishino et al. (65) to only consider unidimensional measurements. In 2017, a consensus guideline for modified RECIST for immune-based therapeutics (termed iRECIST) was published by a multidisciplinary group including academic, commercial and regulatory members for the use of modified RECIST (V1.1) in cancer immunotherapy trials (66). The guideline takes into account distinctive behaviors linked to these types of drugs, such as delayed responses and pseudoprogressions. This guideline is consensus based but is not yet validated. It defines the minimum data to be collected for future and currently in development trials, in order to facilitate the compilation of a data warehouse needed to validate iRECIST. In the meantime, it is recommended that RECIST 1.1 continues to be used as the primary criteria for response based endpoints for randomized studies planned for licensing applications. iRECIST should be considered exploratory in such trials, although earlier phase trials may consider using primarily iRECIST.

Another issue is the delayed treatment effect leading to a separation of PFS or OS curves between treatment arms only after a lag time of several months. This phenomenon has been observed, particularly in melanoma studies (67, 68). This pattern has also been observed for OS in the phase 3 trial comparing nivolumab to standard systemic therapy in patients with recurrent HNSCC (46). Such a late separation is indicative of non-proportional hazards. This pattern may invalidate the use of classical statistical analysis methods to estimate and test treatment effects such as the Cox model, which is based on the assumption of proportional hazards. Such analyses become difficult to interpret since the treatment effect, expressed by the hazard ratio, evolves over time. Alternative analysis methods should therefore be considered and are currently being proposed (63). Models assuming a different hazard ratio for different follow-up times are one possible option. An alternative measure to quantify the treatment effect can be Restricted Mean Survival Time (RMST), which represents the area between the two survival curves up to a predefined follow-up time (69). Simulations are required to evaluate the impact of non-standard patterns on the statistical power using classical or alternative methods of analysis. A delayed treatment effect has also implications on the design of interim analyses for efficacy or futility (63). An interim look for efficacy performed too soon will unlikely result in stopping earlier for a positive outcome, while a futility interim look for futility planned too soon will likely increase the chance of erroneously terminate early the development of an active agent. Altogether this shows how critical is the assumption of proportionality of hazards for the design and analysis of clinical trials with immunotherapy agents.

Although overall survival remains the gold standard endpoint to evaluate the efficacy of treatments in oncology, a number of studies select progression/recurrence free survival as primary endpoint mainly in order to reduce the size and the duration of the studies. As indicated above, it cannot be extrapolated from the work of Michiels et al. that progression-free survival is a surrogate endpoint for overall survival to assess the treatment effect of immunotherapy agents. In addition, as the criteria for progression would be adapted following iRECIST, the issue of surrogacy might be impacted. Surrogate endpoints for immunotherapy trials are currently under investigation (70–72).

### Comorbidities

Comorbidity is frequent in HNSCC patients (73, 74). The main risk factors associated to this cancer are tobacco and alcohol use, so the comorbid illnesses in these patients are largely related to these habits. The most prevalent comorbidities in this population will be cardiovascular, respiratory or neurological affections. Due to their high prevalence of comorbidities head and neck cancer patients are less often included in early phase trials because of their higher risk of complications. Clinical trials severely select patients and requirenormal organ function, whether of the heart, lungs, kidneys, liver or bone marrow at baseline. It is important to bear this in mind when generalizing trials results to the clinical practice population.

#### Impact on Primary Endpoints

Studies evaluating the impact of comorbidities in head and neck cancer patients show that it is an important feature of these patients, which has a detrimental impact on overall survival. Patients with head and neck cancer are concurrently at risk for other events, including second malignancies and mortality due to adverse treatment effects or comorbid diseases (75). Overall survival and progression/recurrence-free survival are composite endpoints, constituted of events of different nature, directly linked to the primary cancer (disease progression/recurrence or death due to the disease) or not (second malignancies, deaths due to treatment toxicity or comorbidities). Analyzed as composite endpoints, they are not sufficient for a complete interpretation of the results of the trial. It is then useful to analyze the components as individual time-to-event endpoint, via cumulative incidence functions, in order to distinguish and characterize the weight of the different components on the observed outcomes.

#### Impact on Adherence to Treatment

Comorbidities have an influence on adherence to planned treatment (treatment missed or delayed), to protocol procedures (e.g., visits missed or delayed) and may induce loss to follow-up. The increasing complexity of treatment strategies and of trial designs with complex protocols which entail multiple procedures, adds an additional layer of difficulty for patients to adhere to treatment and protocol procedures. Oral medications and self-administered subcutaneous therapies offer the patient convenience over intravenous infusions but the responsibility of administration of these critical medications has been transferred to the patient, potentially increasing the risk of non-adherence.

A retrospective analysis of comorbidities and adherence to treatment in patients with oropharyngeal carcinoma has been reported by Hess (76), suggesting a poorer adherence to treatment in patients with HPV-negative status as compared HPV-positive, as a result of the higher comorbidities in the former patient group due to alcohol and tobacco consumption. These results add to the recognition that HPV-positive and HPV-negative oropharyngeal cancer represent distinct entities and the authors recommend to take this additional difference into account in the design of clinical trials addressing these populations.

Poor compliance does affect the analysis and interpretation of clinical trial data and represents a potential source of bias in a clinical trial. The data related to treatment exposure, the frequency and reasons for treatment interruptions or definitive withdrawals, the frequency and nature of severe protocol violations, the frequency of patients lost to follow up need to be analyzed as well as their relationship to outcome in order to identify these potential biases. Sensitivity analyses conducted in different analyses populations, i.e., intention-to-treat vs. per-protocol population, may be useful to assess the robustness of the findings.

#### Impact on Quality of Life and Assessment of Quality of Life in Clinical Trials

The symptoms and treatments associated with advanced head and neck cancer often have a devastating impact on quality of life. Head and neck cancer can disrupt many life essential functions. It can impact on breathing, swallowing, and speaking, and treatment can even increase the physical impairment. These consequences affect multiple spheres of daily functioning. As one consequence head and neck cancer patients have a higher risk of depression and suicide.

Quality of Life is thus an important outcome to be considered in routine treatment but also to evaluate new treatments in clinical trials, even in early stage disease. The EORTC has developed and validated tools for the assessment of quality of life in cancer patients, using high standards of methodology. These questionnaires are meant to be used primarily in clinical trials. Specifically for head and neck cancer, patients are asked to complete a list of 60 head and neck cancer-specific items comprising the recently updated EORTC head and neck module (EORTC QLQ-HN43) as well as the core questionnaire (EORTC QLQ-C30) (77, 78).

For some studies, a quality of life score has been selected as the primary endpoint. This is the case of the EORTC study 1420 “Best-of” (NCT02984410). Because the techniques that have been developed in parallel in the radiotherapy and surgical fields both have an excellent oncological control, the main focus of this prospective randomized trial is to assess which one of the two modalities provides better functional outcome and more specifically better swallowing function. This is assessed using the M. D. Anderson dysphagia inventory (MDADI), a validated and reliable self-administered questionnaire designed specifically for evaluating the impact of dysphagia on the quality of life of patients with head and neck cancer (79).

There is to date no consensus on how quality of life data in cancer clinical trials are analyzed. A variety of statistical techniques are available to handle the longitudinal nature of the data, to adjust for multiple scales and items, to deal with missing data (80). Currently the methods range from simple descriptive analyses up to complex modeling approaches. The consortium SISAQOL (Setting International Standards in Analyzing Patient-Reported Outcomes and Quality of Life Endpoints Data for Cancer Clinical Trials) has been created with the aim to develop guidelines and recommendations for the statistical analyses of quality of life data and more generally of patient-reported outcome data in cancer clinical trials (81, 82).

## Conclusion

The medical statistician is responsible for a wide variety of tasks covering the design and the analysis of a clinical study, which requires specific competencies in terms of statistical methodology and programming skills. It is particularly important to use efficient communication, in order that the medical statistician gets some understanding of the medical field and that the research physician gets fairly acquainted with the principles of statistical methodology. Only a fluid interaction between the two fields enables that the study design addresses adequately the research question that is at the basis of the clinical trial and that the results of the analysis are interpreted appropriately.

In particular head and neck cancer is a complex field: a heterogeneous disease, with multimodality treatment and associated comorbidities. We have set out how these specificities raise a number of methodological challenges with some examples of approaches that current and future clinical researchers and medical statisticians may altogether consider useful in order to generate valuable information to guide clinical decisions and ultimately make progress in the treatment of this disease.

## Author's Note

There is abundant literature in the field of head and neck cancer as well as abundant literature in statistical methodology. The present article makes the bridge between the two fields hopefully encouraging and enabling fluid communication between the research physician and the medical statistician involved in clinical trials in head and neck cancer. The methodological challenges resulting from the heterogeneity of the head and neck cancer, the complexity of the treatments and the associated comorbidities are presented with examples. A formal literature search for this review was not performed. This review is based on the authors' work and expertise in designing, monitoring and analysing clinical trials as well as reading and reviewing clinical and statistical literature. The final purpose of this article is twofold: to help statisticians new to the field of head and neck cancer confronted with the design, implementation and analysis of clinical trials in oncology; but also to sensitize research physicians with the role, the tasks and the challenges faced by the medical statisticians.

## Author Contributions

All authors listed have made a substantial, direct and intellectual contribution to the work, and approved it for publication.

### Conflict of Interest Statement

The authors declare that the research was conducted in the absence of any commercial or financial relationships that could be construed as a potential conflict of interest.
