# Supplementary material for: The Statistical Evaluation of Treatment and Outcomes in Head and Neck Squamous Cell Carcinoma Clinical Trials
Source: Front Oncol. 2019 Jul 12;9:634. doi: 10.3389/fonc.2019.00634 (PMC6640189; doi:10.3389/fonc.2019.00634)
Supplement: Supplementary file 1 [file Table_1.DOCX]

MedLine database was searched using the following Search query:

((((head neck[Title/Abstract] OR oropharynx[Title/Abstract] OR oropharyngeal[Title/Abstract])) AND (cancer[Title/Abstract] OR carcinoma[Title/Abstract])) AND (model[Title/Abstract] OR factor)) AND (prognostic[Title/Abstract] OR predict[Title/Abstract] OR risk classification[Title/Abstract])
